# A Case Report of Autoimmune Haemolytic Anaemia and Acute Hepatitis Associated With Epstein-Barr Virus Infection

**DOI:** 10.7759/cureus.119431

**Published:** 2026-11-10

**Authors:** Eve Wheat

**Affiliations:** 1 General Medicine, Royal Alexandra Hospital, Paisley, GBR

**Keywords:** acute ebv hepatitis, autoimmune haemolytic anaemia, ebv splenomegaly, epstein-barr virus (ebv), new-onset painless jaundice

## Abstract

Infectious mononucleosis is most commonly caused by Epstein-Barr virus (EBV) infection. Although mild hepatic dysfunction is commonly seen, clinically significant acute hepatitis and autoimmune haemolytic anaemia (AIHA) are not seen routinely in clinical practice. The concurrent presentation of both of these poses a diagnostic challenge.

A 22-year-old woman presented with jaundice and fatigue following a recent viral illness; she was found to have significant hepatic dysfunction with a peak bilirubin of 344 µmol/L and haemoglobin nadir of 64 g/L. A positive direct antiglobulin test, with blood film findings, supported the diagnosis of AIHA. EBV serology confirmed acute or recent EBV infection, providing a unifying diagnosis. She was managed supportively with no corticosteroids or blood transfusion, under close monitoring and with advice from the haematology team. Her liver function tests and haemoglobin gradually normalised, and follow-up ultrasound demonstrated resolution of splenomegaly.

This case highlights the importance of considering EBV for a potential underlying, unifying diagnosis for patients presenting with both AIHA and hepatitis, particularly following a viral prodrome and when other causes have been excluded. Ensuring appropriate haematological investigations are undertaken is essential to guide appropriate management and monitoring.

## Introduction

Epstein-Barr virus (EBV) is a member of the herpesvirus family and is extremely common. It is often transmitted through asymptomatic individuals’ saliva; however, it can also be transmitted through sexual contact, solid organ transplantation and blood transfusion [1,2]. There is an incubation period of 4-7 weeks, and an individual can be contagious two weeks prior to the onset of symptoms [2]. Primary infection may result in infectious mononucleosis, which is characterised by a triad of symptoms including sore throat, fever and lymphadenopathy [3]. Hepatic involvement is common although usually subclinical and self-limiting. Abnormal liver function tests, particularly elevated alanine aminotransferase (ALT) levels, are common, whereas significant jaundice or hepatitis is less common [4].

Haemolytic anaemia is a recognised but uncommon complication of EBV infection. Autoantibodies target the surface antigens of erythrocytes. This may result in warm autoimmune haemolytic anaemia (AIHA), which is caused by immunoglobulin G destroying extravascular erythrocytes at a normal body temperature or cold AIHA, which is caused by immunoglobulin M causing complement activation intravascularly at temperatures below 37 degrees Celsius. To diagnose AIHA, a blood film, lactate dehydrogenase (LDH) (released by RBC breakdown), reticulocyte count (raised due to excess bone marrow activity), immunoglobulins, serum serology and direct antiglobulin test (DAT) should be performed. Recognition of AIHA is important as the management of immune-mediated haemolysis differs from other causes of haemolytic anaemia [5].

Jaundice may arise in AIHA through excessive breakdown of erythrocytes resulting in the production of unconjugated bilirubin. However, hepatic injury may impair bilirubin processing and excretion, causing conjugated hyperbilirubinaemia. AIHA and acute hepatitis can occur during EBV infection but AIHA and acute hepatitis occurring concurrently poses a diagnostic challenge [6]. This is why a wide range of differential diagnoses must be taken into account including drug-induced, viral hepatitis and other haematological causes. Establishing EBV as the underlying diagnosis and viral trigger for new-onset haemolytic anaemia and jaundice is important, particularly when there is biochemical evidence of hepatitis. This can avoid more invasive investigations and the initiation of appropriate management of both the haematological and hepatic manifestations. The aim of this case report is to highlight the rare association of EBV infection with concurrent AIHA and acute hepatitis, emphasising the diagnostic challenges of interpreting hyperbilirubinaemia in the presence of both hepatic injury and haemolysis, and the importance of recognising and investigating these coexisting complications.

## Case presentation

A 22-year-old woman attended the medical assessment unit on day 1 following an episode of presyncope, preceded by three days of fever, sore throat, nausea, vomiting and lower abdominal pain. She was generally fit and well and worked as an air hostess. She denied any new medications or supplements and drank minimal alcohol. She was found to have a borderline temperature of 37.9 degrees Celsius, with observations otherwise normal. Her abdomen was soft with mild suprapubic tenderness, and she was jaundiced. Her bloods showed a significant LFT derangement. She was initially reviewed by the surgical team; no surgical cause identified and referred to the medical team. A monospot test was positive, and she was discharged with a plan for repeat blood tests and abdominal ultrasound. 

She re-attended on day 5 with worsening liver function tests (LFTs) and anaemia. Abdominal ultrasound demonstrated splenomegaly, with her spleen measuring 15.7 cm, and the liver, kidneys and gallbladder appeared normal. She was then admitted for further monitoring and investigation. At this point, she reported increased fatigue, a generalised itch and dark urine. She appeared visibly more jaundiced. 

Initial laboratory investigations demonstrated significant hyperbilirubinaemia and deranged LFTs (Table 1). Blood tests showed a mixed hepatic and cholestatic liver injury picture with worsening hyperbilirubinaemia. Total bilirubin 291 umol/L, conjugated 138 umol/L and unconjugated 153 umol/L. Bilirubin increased from 179 umol/L to a peak of 344 umol/L, while ALT peaked at 579 U/L, aspartate aminotransferase (AST) at 535 U/L and alkaline phosphatase (ALP) at 319 U/L. Concurrently, her haemoglobin (Hb) fell from 125 g/L to 64 g/L, with a rising reticulocyte count, supporting ongoing haemolysis. 

**Table 1:** Serial laboratory results from day 1 of presentation to day 35 Serial laboratory results demonstrating an initial rise in bilirubin and liver transaminases followed by a gradual normalisation. Haemoglobin concentrations fall during the initial acute phase of the EBV infection before recovering, consistent with the clinical course of AIHA. (- means test was not completed) EBV: Epstein-Barr virus

	Normal Range	Day 1	Day 2	Day 5	Day 6	Day 7	Day 8	Day 9	Day 10	Day 11	Day 12	Day 13	Day 14	Day 27	Day 35
C-reactive protein (mg/L)	1-10	54	55	21	14	11	-	9	10	8	-	3	-	2	-
Bilirubin (umol/L)	3-21	179	291	306	314	344	262	168	137	112	93	76	68	28	7
AST (U/L)	5-40	182	137	465	535	405	244	183	138	106	79	59	54	18	15
ALT (U/L)	7-50	146	146	430	579	560	435	342	287	246	197	153	133	29	17
ALP (U/L)	30-130	204	227	302	299	319	306	275	277	263	251	215	207	98	81
White cell count (x10^9^/L)	4-11	12.2	11.8	21.8	20	17	19.1	17.3	16.9	14.5	11.8	9.5	7.3	6	6.6
Haemoglobin (g/L)	120-170	125	116	98	84	83	76	67	65	64	68	68	73	114	135
Reticulocytes (x10^9^L)	50-100	94	-	-	-	-	210	-	-	-	-	-	-	101	30

LDH was significantly elevated at 802U/L (reference range: 80-240U/L), haptoglobin was reduced to 0.22 g/L (reference range: 0.3-2.0g/L) and her blood film showed red cell agglutination, strongly suggesting AIHA. The DAT was IgG positive (1+) and C3d strongly positive (3+). A blood film showed normocytic red cells with mature neutrophils and lymphocytes, with occasional reactive forms, in keeping with a viral infection. Other investigations were undertaken for acute liver injury and haemolysis, including viral serology, autoimmune screenings and assessment of haemolytic markers. HIV, CMV and hepatitis A/B/C/E screening were negative. ANA was negative. EBV serology on day 2 demonstrated positive VCA immunoglobulin G and VCA immunoglobulin M, and EBNA immunoglobulin G (EBV) was not detected, supporting acute or recent EBV infection.

Following discussions with haematology, as the patient was asymptomatic, haemodynamically stable and there was no evidence of tissue hypoxia, corticosteroids were not advised, as her haemoglobin was expected to improve with resolution of the acute underlying infection. Transfusion was reserved for symptomatic anaemia. Her haemoglobin and liver biochemistry subsequently improved without specific treatment for the haemolysis including corticosteroids or blood transfusions as she remained asymptomatic of her anaemia. 

A summary of the patient’s course, including key investigations, disease progression and follow-up, is presented in Table 2. By day 14, her bloods were improving, and clinically, she had improved. She was discharged home with a plan for haematology follow-up. In clinic a month later, she was asymptomatic and bloods improving. One month later, her bloods had normalised. Follow-up ultrasound abdomen six months after her admission demonstrated resolution of the splenomegaly, with a spleen measuring 12 cm. She was subsequently discharged from any further haematology follow-up.

**Table 2:** Timeline of clinical presentation, investigations, management and follow-up Summary of the clinical course of the patient including investigations, progression of AIHA and hepatic dysfunction, and then subsequent recovery.

Day	Clinical Events
1	Initial presentation with presyncope, abdominal upset and jaundice. Positive monospot test; discharged with follow-up
5	Readmitted with worsening jaundice, fatigue, pruritus and anaemia. Ultrasound demonstrated splenomegaly (15.7cm) with normal liver outline
7	Peak bilirubin 344 µmol/L
8	Reticulocyte count increased to 210 × 10⁹/L
11	Haemoglobin nadir of 64g/L
14	Clinical and biochemical improvement, discharged with haematology follow-up
27	Haemoglobin improved to 114g/L and bilirubin 28 µmol/L
35	Haemoglobin 135g/L and bilirubin 7µmol/L
6 months	Ultrasound confirmed resolution of splenomegaly (12 cm)

## Discussion

Hepatic involvement is common in acute EBV infection, with 90% of patients having transaminitis; however, clinically significant hepatitis and jaundice are rare [7,8]. The mechanism by which EBV causes hepatitis is not well understood but is thought to be immune-mediated with CD8 T cells rather than direct viral cytotoxicity. CD8 T cells with inflammatory mediators result in hepatocyte injury and hepatic inflammation [8]. The patient's LFTs showed a maximum bilirubin of 344 umol/L, AST 535 U/L, ALT 579 U/L, and ALP 319U/L. In the NICE CKS guidelines, AST and ALT may be 2-3 times the upper limit of normal during the acute phase and normalise within approximately three weeks [9]. The degree of hyperbilirubinaemia and transaminitis in this patient was more marked than the typical transient transaminitis in acute EBV infection. There was no evidence of liver failure in this patient, as her synthetic function remained normal; albumin ranged from 28 to 39 g/L and prothrombin time ratio remained between 1 and 1.2 (normal).

AIHA is a rare, recognised complication of EBV infection; it is caused by an immune-mediated reaction against RBC surface antigens. AIHA can broadly be classified into warm and cold antibody-mediated forms. Warm AIHA is predominantly caused by extravascular destruction of RBCs by IgG autoantibodies, and cold AIHA is usually caused by IgM autoantibodies and complement activation [5]. In this patient, LDH was significantly elevated at 802 U/L, haptoglobin was reduced to 0.22 g/L, and her blood film showed red cell agglutination, strongly suggesting AIHA. The DAT showed IgG positive (1+) and C3D strongly positive (3+). The strong complement activation and red cell agglutination raise the possibility of a mixed or complement-mediated mechanism of haemolysis. However, the weak IgG positivity suggests that the antibody profile was not exclusively characterised by the DAT findings. The cold agglutinin titre, thermal amplitude and antibody specificity tests were not performed; therefore, confirmation of cold AIHA cannot be made. The mechanism of EBV causing AIHA is not fully understood, but the proposed mechanisms include the activation of the complement cascade by molecular mimicry, the anti-EBV antibodies cross-reacting with self-antigens [5]. 

The diagnostic approach in this case was challenging as both hepatitis and AIHA are recognised complications of EBV, and both can present with jaundice and hyperbilirubinaemia, therefore posing a diagnostic challenge and making identifying the underlying cause challenging [5,6]. This means clinicians must distinguish between hepatic dysfunction and haemolysis, and when there is a mixture of both. Therefore, in patients presenting with deranged LFTs, jaundice and evidence of haemolysis, the underlying diagnosis of EBV should be considered. In this case, the combination of significant haemolysis with a haemoglobin dropping from 125 to 64 g/L and hepatitis highlighted the severity of both manifestations and prompted investigation for an underlying potential unifying diagnosis. To identify the cause of jaundice, a broad liver screen (including viral hepatitis, HIV, immunoglobulins and ANA) was sent initially along with an abdominal ultrasound. When she went on to develop haemolysis, further tests including DAT, LDH, reticulocytes and a blood film were performed. EBV was investigated using a monospot test, and then it was confirmed using EBV serology. Following this, EBV confirmed the unifying underlying diagnosis for the hepatitis and AIHA.

This case is similar to previously published reports of EBV-associated haemolytic anaemia. A systematic review of 60 cases of haemolytic anaemia caused by EBV infection found that 52% (N=31) were cold-antibody-mediated mechanisms [10]. This patient had strongly positive C3D and red cell agglutination suggestive of a cold antibody-mediated component; however, further immunohaemotalogical investigations are required to confirm this. Accomando et al. reviewed 16 cases of paediatric AIHA related to EBV and found that the most common associated complications were hepatic injury (53%) [5]. This was demonstrated in this case; this patient was treated supportively with close monitoring. In contrast, the cases reviewed required treatment such as corticosteroids and intravenous immunoglobulin [5].

Acute EBV infection is generally managed using supportive therapies such as analgesia, rest and hydration, typically with symptoms lasting 2-3 weeks. However, it is advised to avoid heavy lifting or contact sports for three weeks following acute infection to reduce the risk of splenic rupture [11]. Hepatitis is typically self-limiting, only requiring monitoring of LFTs, which was the management plan for the patient. AIHA management differs depending on whether the haemolysis is warm- or cold-mediated. Corticosteroids are typically first-line treatment for warm AIHA, and in primary cold AIHA rituximab would be considered if symptomatic. Whereas in secondary cold AIHA, steroids are generally only considered when patients are transfusion dependent, there are severe circulatory issues or symptomatic anaemia. Therefore, management of cold AIHA is focussed on treating the underlying cause, avoiding cold exposure and supportive measures [12]. Therefore, management of cold AIHA is focussed on treating the underlying cause, avoiding cold exposure and supportive measures [11]. As the patient remained clinically stable and asymptomatic throughout despite significant anaemia, they did not require steroid treatment or blood transfusion on advice of the haematology team. Therefore, this patient was managed supportively with close monitoring of her haemoglobin, reticulocytes and LFTs. Ultimately, this patient made a full recovery and was followed up in the haematology clinic on day 35; bloods had normalised and an abdominal ultrasound four months later to assess for splenomegaly showed that the spleen was now 12 cm. 

This patient subsequently improved with no long-term effects, with normalisation of her haemoglobin, bilirubin and LFTs. There was no further evidence of haemolysis and she was discharged from haematology.

A limitation of this case is that further characterisation of the haemolytic mechanism was not undertaken. Therefore, although strong C3D positivity and red cell agglutination supported the diagnosis of complement or mixed AIHA, the precise antibody subtype and mechanism of haemolysis cannot be identified. This case also cannot establish how often this presentation occurs or demonstrate that conservative management would be appropriate for other patients.

## Conclusions

This case highlights the importance of considering EBV infection for the potential unifying diagnosis in patients presenting with AIHA and acute hepatitis, particularly following a viral prodrome. Acute hepatitis with jaundice and AIHA are uncommon in EBV infection; however, mild hepatic dysfunction is commonly seen. Concurrent presentations can pose a diagnostic challenge. Therefore, a thorough investigation into both AIHA and acute hepatitis is required to identify the underlying cause and ensure the appropriate management is initiated. If EBV is recognised early, it may guide further investigations and treatments, leading to earlier management. However, this should not replace a comprehensive diagnostic assessment. This case also highlights that despite severe anaemia and biochemical abnormalities, supportive management and monitoring can result in complete recovery. 
